# Leveraging WeChat for Dementia Caregiver Support: Insights from a Process Evaluation

**DOI:** 10.1002/alz.087671

**Published:** 2025-01-09

**Authors:** Y. Alicia Hong, Kang Shen, Wei‐Hsuan Chang

**Affiliations:** ^1^ George Mason University, Fairfax, VA USA

## Abstract

**Background:**

The evaluation of mHealth interventions often lacks detailed process evaluation data. This study presents the design and results of a process evaluation for the Wellness Enhancement for Caregivers (WECARE) program, an mHealth intervention designed to improve caregiving skills and psychosocial wellbeing of Chinese American dementia caregivers. This evaluation focused on understanding the acceptance, engagement, and the barriers and facilitators influencing behavioral changes among participants, offering valuable feedback for program refinement and dissemination.

**Method:**

A group of 23 Chinese American family caregivers for persons with dementia participated in the WECARE, delivered via WeChat, a widely used social media platform in Mandarin‐speaking communities. WECHAT sent multimedia materials for psychoeducation and skills enhancement six days per week over a seven‐week period. In addition, weekly surveys were distributed via WeChat to gather participants' experiences and suggestions regarding the program.

**Result:**

Response rates to the weekly assessments ranged from 40% to 80%. The study found a higher level of participant interaction with private messaging as opposed to links to external surveys. WeChat’s text and audio message features enriched the quantitative survey data, yielding deeper qualitative insights. The analysis indicated a positive correlation between the applicability of the content and participants' implementation of the intervention strategies in their daily routines, as well as noticeable behavioral improvements. Suggestions from participants were also collected for future enhancements of the program.

**Conclusion:**

The process evaluation data provided critical insights into the implementation and effectiveness of mHealth initiatives, particularly for minority groups such as Chinese American dementia caregivers. The use of short interactive surveys incorporating open‐ended questions on preferred social media platforms proved to be an efficient method for both process evaluation and increasing participant engagement. This study highlights the importance of continued research into process evaluations to refine and improve mHealth interventions for marginalized groups.

